# The untapped potential of phage model systems as therapeutic agents

**DOI:** 10.1093/ve/veae007

**Published:** 2024-01-13

**Authors:** Jordan Romeyer Dherbey, Frederic Bertels

**Affiliations:** Microbial Population Biology, Max Planck Institute for Evolutionary Biology, August-Thienemann-Straße 2, Plön, Schleswig-Holstein 24306, Germany; Microbial Population Biology, Max Planck Institute for Evolutionary Biology, August-Thienemann-Straße 2, Plön, Schleswig-Holstein 24306, Germany

**Keywords:** phage therapy, antibiotic resistance, phage model systems, experimental evolution, ΦX174

## Abstract

With the emergence of widespread antibiotic resistance, phages are an appealing alternative to antibiotics in the fight against multidrug-resistant bacteria. Over the past few years, many phages have been isolated from various environments to treat bacterial pathogens. While isolating novel phages for treatment has had some success for compassionate use, developing novel phages into a general therapeutic will require considerable time and financial resource investments. These investments may be less significant for well-established phage model systems. The knowledge acquired from decades of research on their structure, life cycle, and evolution ensures safe application and efficient handling. However, one major downside of the established phage model systems is their inability to infect pathogenic bacteria. This problem is not insurmountable; phage host range can be extended through genetic engineering or evolution experiments. In the future, breeding model phages to infect pathogens could provide a new avenue to develop phage therapeutic agents.

Infections caused by multidrug-resistant bacterial strains are one of the most pressing issues in medicine, a situation that is only expected to worsen in the coming decades (WHO 2021; Murray et al. 2022). ESKAPEE pathogens (*Enterococcus faecium*, *Staphylococcus aureus*, *Klebsiella pneumoniae*, *Acinetobacter baumannii*, *Pseudomonas aeruginosa*, *Enterobacter* spp., and *Escherichia coli*) are the principal targets for the development of novel antimicrobial strategies (Mulani et al. 2019). Among alternative treatment approaches currently under investigation (e.g. pre- and probiotics, antimicrobial peptides, antibodies, and oligonucleotides for silencing resistance genes), bacteriophages (phages) are one of the most promising alternatives to treat bacterial infections (Rios et al. 2016; Ghosh et al. 2019; Łojewska and Sakowicz 2021; Streicher 2021). Treating bacterial infections with phages is also called phage therapy.

Especially in the past two decades, phage therapy has increasingly been used to treat bacterial infections in humans and animals (Adhya et al. 2005; Maimaiti et al. 2023). Two distinct strategies are commonly followed in phage therapy: a broad and a targeted approach (Gordillo Altamirano and Barr 2019; Froissart and Brives 2021). The broad approach involves assembling a predetermined phage cocktail composed of genetically diverse phages (∼10–40) with a wide host spectrum, emulating the antibiotics’ much broader killing spectrum (Villarroel et al. 2017; McCallin et al. 2018). The targeted approach relies on the use of phages that can specifically infect the bacterial pathogen. These phages are often identified among collections of pre-characterised phages or can be freshly isolated from environmental sources. Phages that demonstrate the best efficiency at infecting and killing the targeting bacterium are administered to the patient (Zhvania et al. 2017; Chan et al. 2018; Ferry et al. 2018; Cano et al. 2020; Dedrick et al. 2021, 2023).

A generic phage cocktail with a broad host spectrum is part of a traditional over-the-counter medicine used in Georgia, Poland, and Russia (McCallin et al. 2018; Międzybrodzki et al. 2018). Vials containing different phage cocktails are sold without a prescription to patients seeking treatment for proinflammatory or enteric diseases (Kutter et al. 2010). The European Union (EU) and United States of America (USA), however, have preferentially developed personalised-medicine approaches that specifically target the pathogen responsible for the bacterial infection (Froissart and Brives 2021). Nonetheless, phage therapy is currently considered highly experimental and can only be used in rare cases as a last resort or compassionate treatment (EMA 2018a; McCallin et al. 2019; FDA 2022; Hitchcock et al. 2023). Compassionate use, also called expanded access, is a treatment option that allows the use of an unauthorised medical product outside clinical trials for the treatment of a patient with a serious or immediately life-threatening disease for which all alternative therapeutic options have been exhausted (EMA 2018a; FDA 2022).

## Advantages and disadvantages of newly isolated phages

Eligible phages for compassionate use come mostly from recent environmental samples. Since the environment is the predominant source of all types of phages, it offers an undeniable advantage to find phages ‘on-demand’ with desired traits for therapeutic purposes (Weber-Dąbrowska et al. 2016; Schooley et al. 2017; Zhvania et al. 2017; Chan et al. 2018; Ferry et al. 2018). Sewage from the immediate vicinity of hospitals is almost guaranteed to contain phages active against human pathogens (Latz et al. 2016). These phages can be easily detected and isolated from environmental samples (Clokie and Kropinski 2009; Ács, Gambino, and Brøndsted 2020), and there is evidence of their efficacy from case studies (McCallin et al. 2019; Abedon, Danis-Wlodarczyk, and Alves 2021). However, isolating phages and generating high-density virus stocks against two of the ESKAPEE species, *E. faecium* and *faecalis* and *S. aureus* strains, have been challenging despite the enormous variety of phages present in environmental reservoirs (Mattila, Ruotsalainen, and Jalasvuori 2015).

The characterisation of new phages from the environment is time-consuming, mainly because of safety and efficacy assessments. Before being considered for clinical applications, a phage’s critical quality attributes (CQAs) must be fully known (Yu et al. 2014; Pirnay et al. 2015; Mutti and Corsini 2019). These include its identity (origin, family and subfamily, morphology, and biology), the presence or absence of potentially damaging genetic determinants (conferring toxicity, virulence, lysogeny, or antibiotic resistance), the phage’s *in vivo* efficacy (host range, stability of lysis, efficiency of plating, and frequency of emergence of phage-resistant bacteria), the potential optimisation of its host range (titration), and its storage conditions (temperature and cryopreservation). Because health agencies require phages to be fully characterised (CQAs) and produced for clinical trials under good manufacturing practices (GMPs), there is currently no broadly available phage treatment in Western countries (Rohde et al. 2018).

GMPs represent the quality, safety, and traceability standards a medicinal product or drug must meet before being authorised for clinical trials and markets (EMA 2018b; Bretaudeau et al. 2020). Phages are categorised as such in the EU and the USA. One exception is Belgium, where phages are produced following a standardised recipe called a monograph (Pirnay et al. 2018). However, the standardisation of phage production requires considerable investment of time and money (Bretaudeau et al. 2020), is difficult to adhere to because of high phage mutation rates (Pirnay et al. 2018), and might be technologically impossible if phages have to be trained to enhance their lytic ability or when phage cocktails are needed to make the treatment resilient against evolution of phage resistances (Yang et al. 2020; Borin et al. 2021; Science, Innovation, and Technology Committee 2023).

## Phage model systems can become promising therapeutic agents

Alongside the use of newly discovered phages for therapy, well-studied phage model systems should also be considered. Model phages such as Dp-1, T4, T7, MS2, or ΦX174 have significant benefits over newly isolated phages.

The main difference between newly isolated phages and model phages is the knowledge available on their biology. The deep knowledge accumulated for model phages should make them predictable and safe therapeutic agents (Bruttin and Brüssow 2005; Wichman, Millstein, and Bull 2005; Bull and Molineux 2008; Budynek et al. 2010; Wichman and Brown 2010; Azam and Tanji 2019). Model phages are easily obtainable, manipulatable, trackable, and producible at high concentrations (Skaradzińska et al. 2020).

Although model phages have not been used in phage therapy yet, they have been used for different clinical applications. For example, model phages have been used as gene delivery vehicles for *in vivo* treatments (Ghaemi et al. 2010; Bakhshinejad and Sadeghizadeh 2014; Fu and Li 2016; Hosseinidoust 2017). The deliveries range from biofilm-degrading enzymes (Lu and Collins 2007) to *in situ* Clustered Regularly Interspaced Short Palindromic Repeats-Cas chromosomal targeted systems (Dong et al. 2021; Huan et al. 2023). These delivery systems have been used for gene therapy and to treat tumours (Ghaemi et al. 2010; Rao and Zhu 2022; Zhu et al. 2023).

While phage vectors could also be created to release antimicrobial compounds *in situ* to treat pathogenic bacterial strains (Du et al. 2023), the possibility of directly turning model phages into the primary therapeutic agent has, to our knowledge, not been investigated (Gildea et al. 2022). Probably because model phages only infect harmless relatives of dangerous pathogenic strains such as *E. coli, Salmonella*, and *Streptococcus* species.

First steps towards extending the host range of model species have been done in the past. For example, Phage T2 has been engineered to infect pathogenic *E. coli* O157:H7 by exchanging phage coat proteins g37 and g38 with those of a newly isolated phage PP01 (Yoichi et al. 2005). In the model Phage T3, host range mutants have been constructed through site-directed mutagenesis that reduced the evolution of phage resistance (Yehl et al. 2019). In microviruses, exchanging coat proteins through genetic engineering successfully extended the host range of Phages ST-1 and α3 (Roznowski et al. 2019).

Genetic engineering has been shown to successfully extend host ranges of model and non-model phages. However, extending the host range via genetic engineering usually involves the transmission of the coat protein of a phage that can infect a target bacterium to another phage of interest (Yoichi et al. 2005; Roznowski et al. 2019). In the absence of phages that can infect a bacterium of interest, evolution experiments are an efficient approach to extend host ranges. Model phages, in particular, could be bred to extend their host range to directly infect pathogenic strains belonging to *E. coli, Salmonella*, and *Streptococcus* species and reduce the evolution of phage resistance (Bull et al. 2003; Meyer et al. 2012; Borin et al. 2021; Romeyer Dherbey et al. 2023). In our opinion, ΦX174 is a particularly interesting model system. We will highlight specific advantages and features of this phage model in the following paragraphs.

## ΦX174 may be a suitable candidate for phage therapy

ΦX174 is one of the oldest phage model systems (Sertic and Bulgakov 1935; Wichman and Brown 2010; Lacković and Toljan 2020) that has been used for almost 90 years to study phage, molecular, synthetic, and evolutionary biology (Sanger et al. 1978; Smith et al. 2003; Jaschke et al. 2012; Mukherjee et al. 2015; Breitbart and Fane 2021). ΦX174 is a small (∼30 nm) tailless coliphage belonging to the *Microviridae* family. It carries a 5,386 nucleotide long single-stranded DNA (ssDNA) genome that contains only eleven genes (Sinsheimer 1959; Sanger et al. 1978). ΦX174 is a virulent phage that relies on attaching to the core oligosaccharide of the host’s lipopolysaccharide (LPS) for infection. In the laboratory, ΦX174 infects—and hence is usually grown on—*E. coli* C, which produces rough type (i.e. lacking the O-antigen) LPS molecules (Feige and Stirm 1976).

The knowledge accumulated on ΦX174 may also be useful to turn ΦX174 into a therapeutic agent. ΦX174 can easily be fully synthesised (Smith et al. 2003) and manipulated in the laboratory (Christakos et al. 2016), making genetic engineering extremely easy. The effect of a large number of mutations and the function of protein domains in the viral life cycle have been studied extensively (Breitbart and Fane 2021). Deep knowledge on the effect of individual mutations may be useful because it could help identify the cause for viral evolution during therapy. Insight into causes for viral evolution will help understand why clinical trials fail to be sufficiently efficacious, the main cause for clinical trial failure at the moment (Hitchcock et al. 2023). Identifying and understanding the cause for treatment failure will make it easier to design efficacious therapeutic agents.

Therapeutic agents have to be both efficacious and safe. Phages are generally considered safe for human application (Liu et al. 2021). Similarly, ΦX174 should be a very safe therapeutic agent. ΦX174 is highly host specific. In a study of 783 different *E. coli* isolates, only six (0.8 per cent) isolates could be infected by ΦX174 (Michel et al. 2010). This high degree of specificity means that ΦX174, like other phages, will likely be harmless to the patient’s microbiota in contrast to antibiotics (Denou et al. 2009; Galtier et al. 2016; Ramirez et al. 2020; Mu et al. 2021). Moreover, relatives of ΦX174, the *Microviridae* phages, can be isolated from gut samples and are considered part of the healthy human gut microbiome (Lim et al. 2015; Manrique et al. 2016; Shkoporov et al. 2019; Sausset et al. 2020). As such, *Microviridae* phages from the gut are probably tolerated by the human immune system and will be less prone to be recognised and degraded prior to successful infection (Hodyra-Stefaniak et al. 2015; Bull, Levin, and Molineux 2019). Evidence for the tolerance of ΦX174 by the immune system without excessive inflammatory response comes from *in vivo* experiments. For those experiments, high doses of ΦX174 were given to patients intravenously to measure differences between healthy individuals and patients with compromised immunity (Ochs, Davis, and Wedgwood 1971; Fogelman et al. 2000). ΦX174 has even been approved for human applications by the U.S. Food and Drug Administration as a marker of patients’ immune responses (Rubinstein et al. 2000; Bearden et al. 2005). While treatment safety is not a major concern for compassionate use cases and phage treatment is generally considered safe, rare side effects could become more of an issue when phages are applied to large parts of the population and over long periods of time.

While ΦX174’s high host specificity reduces potential side effects, it also severely limits ΦX174’s application. However, ΦX174’s limited ability to infect a host can potentially be remedied through evolution experiments. If ΦX174 can infect a host, then it is almost guaranteed to be able to kill it. ΦX174 expresses the E protein to lyse and kill the host by disrupting peptidoglycan synthesis (Orta et al. 2023). Peptidoglycan synthesis is disrupted through binding of the E protein to a very conserved and essential protein called MraY (Bernhardt, Roof, and Young 2000). In biotechnology, the expression of the E protein is used to make ‘ghost cells’ (empty bacterial cell envelopes) for vaccine production. This process works for a wide range of Gram-negative bacterial pathogens (e.g. *Salmonella enteritidis, Vibrio cholera*, and *Helicobacter pylori*) (Huter et al. 1999; Mayr et al. 2005; Ganeshpurkar et al. 2014). Hence, ΦX174 is predicted to be able to lyse any Gram-negative pathogen as long as it can recognise the host’s LPS molecules.

Before a phage infects and kills a host bacterium, it needs to reach it. The smaller the phage, the easier it is for it to diffuse through the medium and reach the target bacterium. ΦX174 is extremely small, and its genome contains only eleven genes. The small size of ΦX174 (100 times smaller than T4, ∼2 million daltons vs 192 million daltons) allows for extremely fast diffusion through media (almost 10 times faster than T4) (Dubin et al. 1970; Bayer and DeBlois 1974). The small genome of ΦX174 also makes it one of the fastest growing phages, producing about 200 offspring per infected cell within 20 min (T4 produces about 80 progeny in 1 h) (Hutchison and Sinsheimer 1966; Eshelman et al. 2010). Both small size and fast replication probably make microviruses one of the most widespread and abundant phage families (Kirchberger and Ochman 2023).

Despite their abundance, no microviruses have so far been considered as the therapeutic agent (Kirchberger, Martinez, and Ochman 2022). One reason for the lack of consideration may be the lack of awareness of their biological importance. Biased isolation and sequencing methods, which failed to identify small ssDNA phages, have wrongly concluded that microviruses are almost non-existent in nature (Kirchberger and Ochman 2023). In contrast, recent research highlights their diversity and abundance in microbiomes (Creasy et al. 2018; Tisza et al. 2020; Zuo et al. 2020).

### Current limitations of ΦX174

The most significant limitation to the current potential of model phages is their host specificity. ΦX174, in particular, is highly host specific (Michel et al. 2010). While this limits possible side effects, no study has yet demonstrated that ΦX174 can infect pathogens. To treat enterobacterial pathogens, novel ΦX174 strains must first be evolved. In previous experiments, we showed that ΦX174 can quickly evolve to infect spontaneously resistant *E. coli* C mutants (Romeyer Dherbey et al. 2023). Whether it is as easy to evolve ΦX174 to infect pathogenic strains remains to be tested.

While its small genome renders ΦX174 extremely tractable for genetic manipulation and analysis, as well as making it extremely unlikely to transport cargo genes, it also means that there is very limited space to easily add useful genes to the genome (Russell and Müller 1984; Aoyama and Hayashi 1985). Phage model systems with bigger genomes can more easily accommodate additional genes.

As with antibiotics, ΦX174 (and most other phages) can infect growing bacteria (Romeyer Dherbey et al. 2023) but cannot infect bacteria in stationary phase or dormancy (Bläsi, Henrich, and Lubitz 1985). Hence, ΦX174 may be more suited to treating acute rather than persistent infections. There are phage model systems that can infect bacteria in stationary phase that, in some situations, may be more appropriate therapeutic agents (Bryan et al. 2016; Tabib-Salazar et al. 2018; Kaldalu et al. 2020; La Rosa et al. 2021; Maffei et al. 2022).

For pathogens other than *E. coli* or *Salmonella*, ΦX174 may also not be the ideal model system. Beyond enterobacterial infections, novel phage model systems need to be established to treat other members of the ESKAPEE group, especially for *A. baumannii*, *E. faecium*, and *S. aureus* (Mattila, Ruotsalainen, and Jalasvuori 2015).

## Evolving phages to infect bacterial pathogens

**Figure 1.**
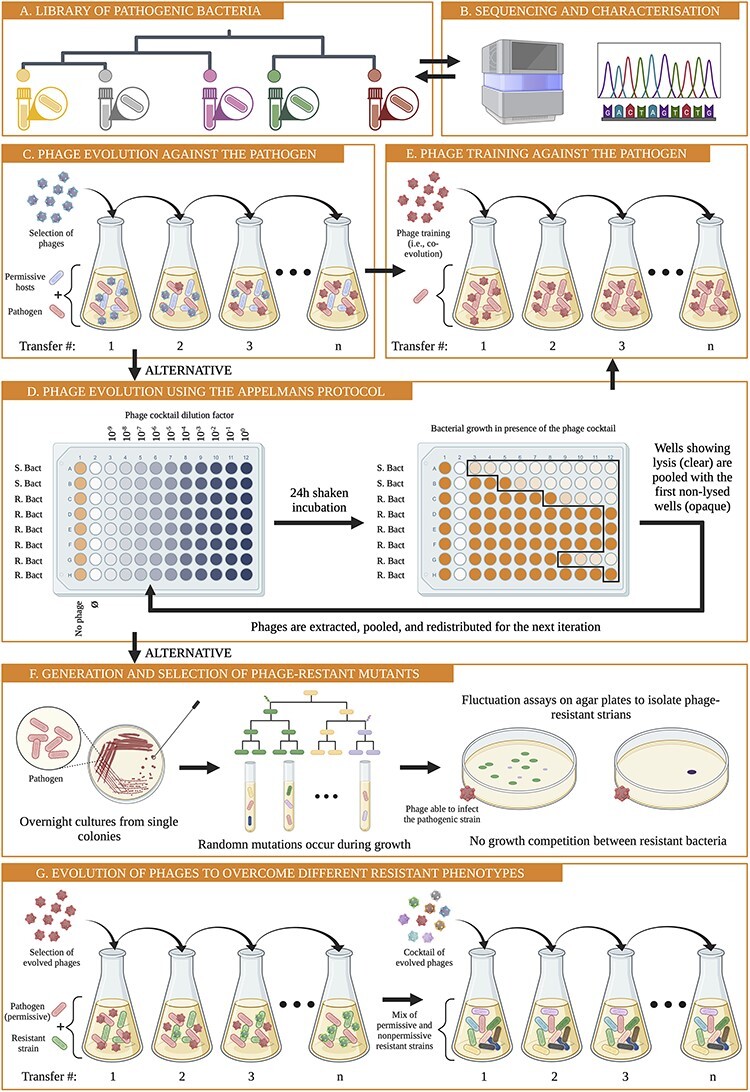
The proposed procedure to develop a phage model system into a therapeutic agent. (A) and (B) Bacterial pathogens are first sequenced and characterised. Phylogenetic trees can help to identify bacterial strains closely related to the target pathogen. Model phages are then adapted to the bacterial pathogens as well as closely related strains *in vitro*. (C) A selection of phages is pooled and serially transferred daily on a host culture containing a mixture of susceptible strains and the pathogenic strain of interest. Transfers continue until a phage is found to infect the pathogenic strain (Bono et al. 2013; Romeyer Dherbey et al. 2023). (D) Phage host range can also be increased using the Appelmans protocol. A selection of phages is pooled and iteratively grown on permissive and non-permissive bacterial strains. To maintain phage diversity from one iteration to another, the first rows contain permissive strains, followed by resistant pathogenic strains. Adapted from Burrowes, Molineux, and Fralick (2019). (E) Phages capable of infecting the pathogenic strains can be further trained to enhance their lytic ability against the pathogen, for example, by phage training in a coevolution experiment (Borin et al. 2021) or through a more targeted approach (F and G) (Romeyer Dherbey et al. 2023). (F) Emergence of phage resistance can be reduced by evolving a range of phage mutants that can infect spontaneously resistant bacteria. Spontaneous phage-resistant mutants can be generated on agar plates using a fluctuation assay (Luria and Delbrück 1943). (G) Left panel: similar to panel (C), phage strains are evolved to infect different phage-resistant variants without coevolution of the bacteria (Romeyer Dherbey et al. 2023). Right panel: for resistant strains that are difficult to infect, additional evolution experiments using a cocktail of phages adapted to easier resistant phenotypes (resistant phenotypes that phages evolved to infect quickly) may speed up evolution via recombination. Host diversity can help maintain phage diversity in the experiment (Romeyer Dherbey et al. 2023).

To develop ΦX174 (and other model phages) into a therapeutic agent that infects pathogens, existing experimental evolution protocols can be adapted (Bono et al. 2013; Burrowes, Molineux, and Fralick 2019; Kok et al. 2023; Romeyer Dherbey et al. 2023) (Fig. 1). Firstly, the bacterial pathogen and several closely related strains need to be isolated and characterised (Fig. 1A and B). Then, a phage strain with the capacity to infect the pathogenic strain is evolved by serially transferring candidate phages in a mixture consisting of permissive hosts (necessary to propagate the phage) and the targeted pathogenic strain (Fig. 1C). Evolving phage populations are inoculated into fresh, exponentially growing host cultures at each transfer until one or more phages are found to infect the pathogenic strain.

Alternatively, the host range of model phages can be extended using the Appelmans protocol (Burrowes, Molineux, and Fralick 2019). This experimental evolution protocol is highly effective at increasing phage host ranges by maximising the recombination opportunities between phage strains (Fig. 1D). It has also been used to enhance the infectivity of phages, thus making phages more effective therapeutic agents (Kok et al. 2023).

A successful therapeutic agent also needs to minimise the chance of phage resistance evolution. Phage resistance evolution can be minimised by combining phages with antibiotics or by combining different phages in cocktails. A phage cocktail aims to eliminate common bacterial resistance types and drive evolution toward bacterial mutants that are less fit and easier to eradicate (Yethon et al. 2000; Matsuura 2013; Pagnout et al. 2019; Simpson and Trent 2019; Burmeister et al. 2020; Mutalik et al. 2020). The immune system and/or specific antibiotics could then kill the remaining mutants (Roach et al. 2017; Burmeister et al. 2020; Mangalea, Duerkop, and Ottemann 2020). Phage resistance evolution can also be lowered by subinhibitory levels of antibiotics. In this case, the antibiotics prevent the emergence of a specific set of bacterial mutants (Parab et al. 2023).

Phage cocktails can consist of distantly related phages or of phages that are derived from a recent common ancestor. These recently diverged phages can be evolved through coevolution experiments, also called phage training (Borin et al. 2021) (Fig. 1E). Coevolution means that both phages and bacteria evolve at the same time and place. When cultures are transferred into fresh media, both evolved phages and evolved bacteria are transferred together. An alternative evolution experiment applies a more targeted approach, where bacteria and phages evolve sequentially. In the sequential evolution approach, phage-resistant mutants are first generated in fluctuation experiments (Luria and Delbrück 1943; Burmeister et al. 2020; Romeyer Dherbey et al. 2023) (Fig. 1F). New phage strains are then evolved to infect each resistant mutant (Fig. 1G). Finally, a selection of the evolved phages can be combined to create an effective phage cocktail (Yehl et al. 2019; Yang et al. 2020; Nale et al. 2021). Phages in these cocktails cannot only infect a diverse set of resistant bacterial strains but also recombine both *in vitro* and *in vivo* to generate phages that can infect bacteria with novel resistance phenotypes (De Sordi, Khanna, and Debarbieux 2017; Burrowes, Molineux, and Fralick 2019; Borin et al. 2021; Srikant, Guegler, and Laub 2022; Romeyer Dherbey et al. 2023).

The sequential evolution approach is likely more laborious than the coevolutionary approach since the bacterium can become phage resistant through many different pathways. However, knowledge about the identity and order of mutations makes it easier to understand how phage resistance works and how phages can overcome different types of resistance. A deeper understanding of phage resistance mechanisms will also make the application of synthetic approaches more effective.

The ability of phages to infect a host is critically dependent on the environment (Kim and Kathariou 2009; Koskella and Brockhurst 2014; Hernandez and Koskella 2019). Hence, once model phages have been evolved to infect pathogens *in vitro*, they may also have to be tested and potentially adapted to *in vivo* conditions before they can be used as therapeutic agents (De Sordi, Lourenço, and Debarbieux 2018; Hernandez and Koskella 2019; Hsu et al. 2019; Castledine et al. 2022). For example, bacteria susceptible to phages in solid media may be resistant to phage infection in liquid media (Romeyer Dherbey 2023). Again, experimental evolution may be the perfect tool to either adapt phages to the host environment or evolve phages that are robust to environmental change.

## Raising phage therapy awareness with established phage model systems

Phage therapy has the potential to significantly improve treatment outcomes. However, one crucial aspect that hinges on its success is often overlooked: the perception of the general public. To engage people with phage therapy, we must ensure effective communication about phage research, its current limitations, and, most importantly, its potential to save lives (Gordillo Altamirano and Barr 2019; Ji and Cheng 2021; Niang et al. 2021).

Medical innovations are often met with great scepticism, especially by the general public (Johnson et al. 2020; Barrett et al. 2022). For example, the acceptance of the new messenger RNA Coronavirus Disease (COVID)-19 vaccine has been hampered by the spread of misleading or false information (Hussain et al. 2018; Burki 2020; Longhi 2022). As phages are also viruses, their acceptance and the willingness of people to rely on them as therapeutic agents could be impeded in similar ways. Moreover, phage therapy has already had to overcome the poor reputation obtained through its association with Axis powers during the Second World War and Cold War (Summers 2012). To prevent history from repeating itself, the narrative around phage therapy and its anthropological impact on modern society should be taken into consideration by scientists (biologists, anthropologists of sciences, and sociologists), media, and politics.

Fortunately, we still have time to effectively and transparently communicate about the advantages and limitations of phage therapy. Phage model systems represent a convenient tool for this endeavour as we can capitalise on our profound insight into their biology and evolution (Luciano, Young, and Patterson 2002; Hanauer et al. 2017). The knowledge acquired about model phage systems over the last 100 years will facilitate the communication of complex concepts about phages to the general public. For example, Phage T4 is already used in television reports and science cartoons (Kurzgesagt 2018) as the ‘default phage’, thanks to its striking morphology. Similarly, other phage model systems could be exploited to communicate information on phage biology and phage therapy. Finally, integrating phage biology and phage hunt classes (i.e. phage discovery programmes) may be a good way to construct collective knowledge and disseminate accurate information about phages (Elbers and Streefland 2000; Staub et al. 2016; Hanauer et al. 2017).

## Conclusion

Established phage model systems are far from old fashioned. In addition to the purely economical, biological, and medicinal advantages, they may provide non-negligible sociological benefits. These advantages could be decisive in establishing phage therapy as a common, safe, and inexpensive medical practice in the West once the technology is readily available. Extensive research, however, has first to be conducted to demonstrate the efficacy of phage model systems to treat infection caused by pathogenic bacteria. Hence, in parallel with the ongoing search for novel environmental phages, we advocate investing resources into developing phage model systems for phage therapies.

## References

[R1] Abedon S. T., Danis-Wlodarczyk K. M., and Alves D. R. (2021) ‘Phage Therapy in the 21st Century: Is There Modern, Clinical Evidence of Phage-Mediated Efficacy?’, *Pharmaceuticals (Basel, Switzerland)*, 14: 1157. 10.3390/ph14111157 PMC8625828 34832939

[R2] Ács N., Gambino M., and Brøndsted L. (2020) ‘Bacteriophage Enumeration and Detection Methods’, *Frontiers in Microbiology*, 11: 594868. 10.3389/fmicb.2020.594868 PMC7644846 33193274

[R3] Adhya S. et al. (2005) ‘2004 ASM Conference on the New Phage Biology: The “Phage Summit”’, *Molecular Microbiology*, 55: 1300–14. 15720541 10.1111/j.1365-2958.2005.04509.x

[R4] Aoyama A., and Hayashi M. (1985) ‘Effects of Genome Size on Bacteriophage Phi X174 DNA Packaging *in Vitro*’, *Journal of Biological Chemistry*, 260: 11033–8. 3161888

[R5] Azam A. H., and Tanji Y. (2019) ‘Bacteriophage-Host Arm Race: An Update on the Mechanism of Phage Resistance in Bacteria and Revenge of the Phage with the Perspective for Phage Therapy’, *Applied Microbiology and Biotechnology*, 103: 2121–31. 30680434 10.1007/s00253-019-09629-x

[R6] Bakhshinejad B., and Sadeghizadeh M. (2014) ‘Bacteriophages as Vehicles for Gene Delivery into Mammalian Cells: Prospects and Problems’, *Expert Opinion on Drug Delivery*, 11: 1561–74. 24955860 10.1517/17425247.2014.927437

[R7] Barrett J. S. et al. (2022) ‘Considerations for Addressing Anti-Vaccination Campaigns: How Did We Get Here and What Can We Do about It?’, *Clinical and Translational Science*, 15: 1380–6. 35320620 10.1111/cts.13273 PMC9111546

[R8] Bayer M. E., and DeBlois R. W. (1974) ‘Diffusion Constant and Dimension of Bacteriophage φX174 as Determined by Self-Beat Laser Light Spectroscopy and Electron Microscopy’, *Journal of Virology*, 14: 975–80. 4138908 10.1128/jvi.14.4.975-980.1974 PMC355605

[R9] Bearden C. M. et al. (2005) ‘Rituximab Inhibits the *in Vivo* Primary and Secondary Antibody Response to a Neoantigen, Bacteriophage PhiX174’, *American Journal of Transplantation*, 5: 50–7. 15636611 10.1111/j.1600-6143.2003.00646.x

[R10] Bernhardt T. G., Roof W. D., and Young R. (2000) ‘Genetic Evidence that the Bacteriophage ΦX174 Lysis Protein Inhibits Cell Wall Synthesis’, *Proceedings of the National Academy of Sciences of the United States of America*., 97: 4297–302. 10760296 10.1073/pnas.97.8.4297 PMC18234

[R11] Bläsi U., Henrich B., and Lubitz W. (1985) ‘Lysis of *Escherichia Coli* by Cloned Phi X174 Gene E Depends on Its Expression’, *Journal of General Microbiology*, 131: 1107–14. 3160821 10.1099/00221287-131-5-1107

[R12] Bono L. M. et al. (2013) ‘Competition and the Origins of Novelty: Experimental Evolution of Niche-Width Expansion in a Virus’, *Biology Letters*, 9: 20120616. 10.1098/rsbl.2012.0616 PMC3565482 23075527

[R13] Borin J. M. et al. (2021) ‘Coevolutionary Phage Training Leads to Greater Bacterial Suppression and Delays the Evolution of Phage Resistance’, *Proceedings of the National Academy of Sciences*, 118: e2104592118. 10.1073/pnas.2104592118 PMC8201913 34083444

[R14] Breitbart M., and Fane B. A. (2021) *Microviridae*. In: eLS, pp. 1–14. John Wiley & Sons, Ltd.

[R15] Bretaudeau L. et al. (2020) ‘Good Manufacturing Practice (GMP) Compliance for Phage Therapy Medicinal Products’, *Frontiers in Microbiology*, 11: 1161. 10.3389/fmicb.2020.01161 PMC7287015 32582101

[R16] Bruttin A., and Brüssow H. (2005) ‘Human Volunteers Receiving *Escherichia coli* Phage T4 Orally: A Safety Test of Phage Therapy’, *Antimicrobial Agents and Chemotherapy*, 49: 2874–8. 15980363 10.1128/AAC.49.7.2874-2878.2005 PMC1168693

[R17] Bryan D. et al. (2016) ‘Bacteriophage T4 Infection of Stationary Phase *E. coli*: Life after Log from a Phage Perspective.’, *Frontiers in Microbiology*, 7: 1391. 10.3389/fmicb.2016.01391 PMC5014867 27660625

[R18] Budynek P. et al. (2010) ‘Bacteriophages and Cancer’, *Archives of Microbiology*, 192: 315–20. 20232198 10.1007/s00203-010-0559-7

[R19] Bull J. J. et al. (2003) ‘Experimental Evolution Yields Hundreds of Mutations in a Functional Viral Genome’, *Journal of Molecular Evolution*, 57: 241–8. 14629033 10.1007/s00239-003-2470-1

[R20] Bull J. J., Levin B. R., and Molineux I. J. (2019) ‘Promises and Pitfalls of *in Vivo* Evolution to Improve Phage Therapy’, *Viruses*, 11: 1083. 10.3390/v11121083 PMC6950294 31766537

[R21] Bull J. J., and Molineux I. J. (2008) ‘Predicting Evolution from Genomics: Experimental Evolution of Bacteriophage T7’, *Heredity*, 100: 453–63. 18212807 10.1038/sj.hdy.6801087

[R22] Burki T. (2020) ‘The Online Anti-Vaccine Movement in the Age of COVID-19’, *The Lancet Digital Health*, 2: e504–5. 32984795 10.1016/S2589-7500(20)30227-2 PMC7508526

[R23] Burmeister A. R. et al. (2020) ‘Pleiotropy Complicates a Trade-Off between Phage Resistance and Antibiotic Resistance’, *Proceedings of the National Academy of Sciences*, 117: 11207–16. 10.1073/pnas.1919888117 PMC7260982 32424102

[R24] Burrowes B., Molineux I., and Fralick J. (2019) ‘Directed *in Vitro* Evolution of Therapeutic Bacteriophages: The Appelmans Protocol’, *Viruses*, 11: 241. 10.3390/v11030241 PMC6466182 30862096

[R25] Cano E. J. et al. (2020) ‘Phage Therapy for Limb-Threatening Prosthetic Knee *Klebsiella pneumoniae* Infection: Case Report and *in Vitro* Characterization of Anti-Biofilm Activity’, *Clinical Infectious Diseases*, 73: e144–51. 10.1093/cid/ciaa705 PMC8246933 32699879

[R26] Castledine M. et al. (2022) ‘Parallel Evolution of *Pseudomonas aeruginosa* Phage Resistance and Virulence Loss in Response to Phage Treatment *in Vivo* and *in Vitro*’, *eLife*, 11: e73679. 10.7554/eLife.73679 PMC8912922 35188102

[R27] Chan B. K. et al. (2018) ‘Phage Treatment of an Aortic Graft Infected with *Pseudomonas aeruginosa*’, *Evolution, Medicine, and Public Health*, 2018: 60–6. 29588855 10.1093/emph/eoy005 PMC5842392

[R28] Christakos K. J. et al. (2016) ‘PhiXing-it, Displaying Foreign Peptides on Bacteriophage ΦX174’, *Virology*, 488: 242–8. 26655242 10.1016/j.virol.2015.11.021 PMC6191337

[R29] Clokie, M. R. J., and Kropinski, A. M. (eds.) (2009) *Bacteriophages: Methods and Protocols*. Humana Press: New York.

[R30] Creasy A. et al. (2018) ‘Unprecedented Diversity of ssDNA Phages from the Family Microviridae Detected within the Gut of a Protochordate Model Organism (Ciona robusta)’, *Viruses*, 10: 404. 10.3390/v10080404 PMC6116155 30065169

[R31] Dedrick R. M. et al. (2021) ‘Potent Antibody-Mediated Neutralization Limits Bacteriophage Treatment of a Pulmonary *Mycobacterium abscessus* Infection’, *Nature Medicine*, 27: 1357–61. 10.1038/s41591-021-01403-9 PMC8571776 34239133

[R32] Dedrick R. M. et al. (2023) ‘Phage Therapy of *Mycobacterium* Infections: Compassionate Use of Phages in 20 Patients with Drug-Resistant Mycobacterial Disease’, *Clinical Infectious Diseases*, 76: 103–12. 35676823 10.1093/cid/ciac453 PMC9825826

[R33] Denou E. et al. (2009) ‘T4 Phages against *Escherichia coli* Diarrhea: Potential and Problems’, *Virology*, 388: 21–30. 19339031 10.1016/j.virol.2009.03.009

[R34] De Sordi L., Khanna V., and Debarbieux L. (2017) ‘The Gut Microbiota Facilitates Drifts in the Genetic Diversity and Infectivity of Bacterial Viruses’, *Cell Host & Microbe*, 22: 801–8.e3. 29174401 10.1016/j.chom.2017.10.010

[R35] De Sordi L., Lourenço M., and Debarbieux L. (2018) ‘“I Will Survive”: A Tale of Bacteriophage-Bacteria Coevolution in the Gut’, *Gut Microbes*, 10: 92–9. 29913091 10.1080/19490976.2018.1474322 PMC6363074

[R36] Dong J. et al. (2021) ‘Engineering T4 Bacteriophage for *in Vivo* Display by Type V CRISPR-Cas Genome Editing.’, *ACS Synthetic Biology*, 10: 2639–48. 34546037 10.1021/acssynbio.1c00251 PMC12867177

[R37] Du J. et al. (2023) ‘Enhancing Bacteriophage Therapeutics through in Situ Production and Release of Heterologous Antimicrobial Effectors’, *Nature Communications*, 14: 4337. 10.1038/s41467-023-39612-0 PMC10359290 37474516

[R38] Dubin S. B. et al. (1970) ‘Molecular Weights of Coliphages and Coliphage DNA: II. Measurement of Diffusion Coefficients Using Optical Mixing Spectroscopy, and Measurement of Sedimentation Coefficients’, *Journal of Molecular Biology*, 54: 547–56. 5492019 10.1016/0022-2836(70)90125-7

[R39] Elbers E., and Streefland L. (2000) ‘Collaborative Learning and the Construction of Common Knowledge’, *European Journal of Psychology of Education*, 15: 479–90.

[R40] EMA (2018a), Compassionate Use (European Medicines Agency) <https://www.ema.europa.eu/en/human-regulatory/research-development/compassionate-use> accessed 24 Jan 2024.

[R53] ——— (2018b), Good Manufacturing Practice ( European Medicines Agency) <https://www.ema.europa.eu/en/human-regulatory/research-development/compliance/good-manufacturing-practice> accessed 24 Jan 2024.

[R41] Eshelman C. M. et al. (2010) ‘Unrestricted Migration Favours Virulent Pathogens in Experimental Metapopulations: Evolutionary Genetics of a Rapacious Life History’, *Philosophical Transactions of the Royal Society B: Biological Sciences*, 365: 2503–13. 10.1098/rstb.2010.0066 PMC2935104 20643740

[R42] FDA . (2022), Expanded Access (U.S. Food and Drug Administration) <https://www.fda.gov/news-events/public-health-focus/expanded-access> accessed 24 Jan 2024.

[R43] Feige U., and Stirm S. (1976) ‘On the Structure of *Escherichia coli* C Cell Wall Lipopolysaccharide Core and Its ΦX174 Receptor Region’, *Biochemical and Biophysical Research Communications*, 71: 566–73. 786289 10.1016/0006-291x(76)90824-x

[R44] Ferry T. et al. (2018) ‘Innovations for the Treatment of a Complex Bone and Joint Infection Due to XDR *Pseudomonas aeruginosa* Including Local Application of a Selected Cocktail of Bacteriophages’, *Journal of Antimicrobial Chemotherapy*, 73: 2901–3. 30060002 10.1093/jac/dky263

[R45] Fogelman I. et al. (2000) ‘Evaluation of CD4 + T Cell Function *in Vivo* in HIV-Infected Patients as Measured by Bacteriophage PhiX174 Immunization’, *The Journal of Infectious Diseases*, 182: 435–41. 10915073 10.1086/315739

[R46] Froissart R., and Brives C. (2021) ‘Evolutionary Biology and Development Model of Medicines: A Necessary “Pas de Deux” for Future Successful Bacteriophage Therapy’, *Journal of Evolutionary Biology*, 34: 1855–66. 34288190 10.1111/jeb.13904

[R47] Fu Y., and Li J. (2016) ‘A Novel Delivery Platform Based on Bacteriophage MS2 Virus-Like Particles’, *Virus Research*, 211: 9–16. 26415756 10.1016/j.virusres.2015.08.022 PMC7114531

[R48] Galtier M. et al. (2016) ‘Bacteriophages to Reduce Gut Carriage of Antibiotic Resistant Uropathogens with Low Impact on Microbiota Composition’, *Environmental Microbiology*, 18: 2237–45. 26971586 10.1111/1462-2920.13284

[R49] Ganeshpurkar A. et al. (2014) ‘Harnessing the Potential of Bacterial Ghost for the Effective Delivery of Drugs and Biotherapeutics’, *International Journal of Pharmaceutical Investigation*, 4: 1. 10.4103/2230-973X.127733 PMC3944611 24678455

[R50] Ghaemi A. et al. (2010) ‘Recombinant λ-Phage Nanobioparticles for Tumor Therapy in Mice Models’, *Genetic Vaccines and Therapy*, 8: 3. 10.1186/1479-0556-8-3 PMC2890663 20459865

[R51] Ghosh C. et al. (2019) ‘Alternatives to Conventional Antibiotics in the Era of Antimicrobial Resistance’, *Trends in Microbiology*, 27: 323–38. 30683453 10.1016/j.tim.2018.12.010

[R52] Gildea L. et al. (2022) ‘P22 Phage Shows Promising Antibacterial Activity under Pathophysiological Conditions’, *Archives of Microbiology & Immunology*, 6: 81–100. 35996377 10.26502/ami.93650078 PMC9392898

[R54] Gordillo Altamirano F. L., and Barr J. J. (2019) ‘Phage Therapy in the Postantibiotic Era’, *Clinical Microbiology Reviews*, 32: e00066–18. 30651225 10.1128/CMR.00066-18 PMC6431132

[R55] Hanauer D. I. et al. (2017) ‘An Inclusive Research Education Community (iREC): Impact of the SEA-PHAGES Program on Research Outcomes and Student Learning’, *Proceedings of the National Academy of Sciences*, 114: 13531–6. 10.1073/pnas.1718188115 PMC5754813 29208718

[R56] Hernandez C. A., and Koskella B. (2019) ‘Phage Resistance Evolution *in Vitro* Is Not Reflective of *in Vivo* Outcome in a Plant-Bacteria-Phage System’, *Evolution*, 73: 2461–75. 31433508 10.1111/evo.13833

[R57] Hitchcock N. M. et al. (2023) ‘Current Clinical Landscape and Global Potential of Bacteriophage Therapy’, *Viruses*, 15: 1020. 10.3390/v15041020 PMC10146840 37113000

[R58] Hodyra-Stefaniak K. et al. (2015) ‘Mammalian Host-versus-Phage Immune Response Determines Phage Fate *in Vivo*’, *Scientific Reports*, 5: 14802. 10.1038/srep14802 PMC4594097 26440922

[R59] Hosseinidoust Z. (2017) ‘Phage-Mediated Gene Therapy’, *Current Gene Therapy*, 17: 120–6. 28494733 10.2174/1566523217666170510151940

[R60] Hsu B. B. et al. (2019) ‘Dynamic Modulation of the Gut Microbiota and Metabolome by Bacteriophages in a Mouse Model’, *Cell Host & Microbe*, 25: 803–14.e5. 31175044 10.1016/j.chom.2019.05.001 PMC6579560

[R61] Huan Y. W. et al. (2023) ‘P1 Bacteriophage-Enabled Delivery of CRISPR-Cas9 Antimicrobial Activity against *Shigella flexneri*.’, *ACS Synthetic Biology*, 12: 709–21. 36802585 10.1021/acssynbio.2c00465 PMC10028697

[R62] Hussain A. et al. (2018) ‘The Anti-Vaccination Movement: A Regression in Modern Medicine’, *Cureus*, 10: e2919. 10.7759/cureus.2919 PMC6122668 30186724

[R63] Hutchison C. A., and Sinsheimer R. L. (1966) ‘The Process of Infection with Bacteriophage ΦX174: X. Mutations in a ΦX Lysis Gene’, *Journal of Molecular Biology*, 18: 429–47. 5968177 10.1016/s0022-2836(66)80035-9

[R64] Huter V. et al. (1999) ‘Bacterial Ghosts as Drug Carrier and Targeting Vehicles’, *Journal of Controlled Release*, 61: 51–63. 10469902 10.1016/s0168-3659(99)00099-1

[R65] Jaschke P. R. et al. (2012) ‘A Fully Decompressed Synthetic Bacteriophage ΦX174 Genome Assembled and Archived in Yeast’, *Virology*, 434: 278–84. 23079106 10.1016/j.virol.2012.09.020

[R66] Ji R., and Cheng Y. (2021) ‘Thinking Global Health from the Perspective of Anthropology’, *Global Health Research and Policy*, 6: 1–3. 10.1186/s41256-021-00233-z PMC8636067 34852852

[R67] Johnson N. F. et al. (2020) ‘The Online Competition between Pro- and Anti-Vaccination Views’, *Nature*, 582: 230–3. 32499650 10.1038/s41586-020-2281-1

[R68] Kaldalu N. et al. (2020) ‘*In Vitro* Studies of Persister Cells’, *Microbiology and Molecular Biology Reviews*, 84: e00070–20. 10.1128/MMBR.00070-20 PMC7667008 33177189

[R69] Kim J.-W., and Kathariou S. (2009) ‘Temperature-Dependent Phage Resistance of *Listeria monocytogenes* Epidemic Clone II’, *Applied and Environmental Microbiology*, 75: 2433–8. 19251898 10.1128/AEM.02480-08 PMC2675217

[R70] Kirchberger P. C., Martinez Z. A., and Ochman H. (2022) ‘Organizing the Global Diversity of Microviruses’, *mBio*, 13: e00588–22. 35491833 10.1128/mbio.00588-22 PMC9239249

[R71] Kirchberger P. C., and Ochman H. (2023) ‘Microviruses: A World beyond PhiX174’, *Annual Review of Virology*, 10: 99–118. 10.1146/annurev-virology-100120-011239 37774127

[R72] Kok D. N. et al. (2023) ‘*In Vitro* Evolution to Increase the Titers of Difficult Bacteriophages: RAMP-UP Protocol’, *PHAGE*, 4: 68–81. 37350994 10.1089/phage.2023.0005 PMC10282794

[R73] Koskella B., and Brockhurst M. A. (2014) ‘Bacteria–Phage Coevolution as a Driver of Ecological and Evolutionary Processes in Microbial Communities’, *FEMS Microbiology Reviews*, 38: 916–31. 24617569 10.1111/1574-6976.12072 PMC4257071

[R74] Kurzgesagt . (2018), The Deadliest Being on Planet Earth—the Bacteriophage—YouTube. <https://www.youtube.com/watch?v=YI3tsmFsrOg> accessed 24 Jan 2024.

[R75] Kutter E. et al. (2010) ‘Phage Therapy in Clinical Practice: Treatment of Human Infections’, *CDATA (Current Pharmaceutical Biotechnology)*, 11: 69–86. 10.2174/138920110790725401 20214609

[R76] Lacković Z., and Toljan K. (2020) ‘Vladimir Sertić: Forgotten Pioneer of Virology and Bacteriophage Therapy’, *Notes and Records of the Royal Society of London*, 74: 567–78. 33177747 10.1098/rsnr.2019.0010 PMC7653334

[R77] La Rosa R. et al. (2021) ‘Compensatory Evolution of *Pseudomonas aeruginosa*’s Slow Growth Phenotype Suggests Mechanisms of Adaptation in Cystic Fibrosis’, *Nature Communications*, 12: 3186. 10.1038/s41467-021-23451-y PMC8160344 34045458

[R78] Latz S. et al. (2016) ‘Preliminary Survey of Local Bacteriophages with Lytic Activity against Multi-Drug Resistant Bacteria’, *Journal of Basic Microbiology*, 56: 1117–23. 27194637 10.1002/jobm.201600108

[R79] Lim E. S. et al. (2015) ‘Early Life Dynamics of the Human Gut Virome and Bacterial Microbiome in Infants’, *Nature Medicine*, 21: 1228–34. 10.1038/nm.3950 PMC4710368 26366711

[R80] Liu D. et al. (2021) ‘The Safety and Toxicity of Phage Therapy: A Review of Animal and Clinical Studies’, *Viruses*, 13: 1268. 10.3390/v13071268 PMC8310247 34209836

[R81] Łojewska E., and Sakowicz T. (2021) ‘An Alternative to Antibiotics: Selected Methods to Combat Zoonotic Foodborne Bacterial Infections’, *Current Microbiology*, 78: 4037–49. 34626217 10.1007/s00284-021-02665-9 PMC8595143

[R82] Longhi J. (2022) ‘The Parascientific Communication around Didier Raoult’s Expertise and the Debates in the Media and on Digital Social Networks during the COVID-19 Crisis in France’, *Publications*, 10: 7.

[R83] Luciano C. S., Young M. W., and Patterson R. R. (2002) ‘Bacteriophage: A Model System for Active Learning’, *Microbiology Education*, 3: 1–6. 23653543 10.1128/me.3.1.1-6.2002 PMC3633122

[R84] Lu T. K., and Collins J. J. (2007) ‘Dispersing Biofilms with Engineered Enzymatic Bacteriophage’, *Proceedings of the National Academy of Sciences*, 104: 11197–202. 10.1073/pnas.0704624104 PMC1899193 17592147

[R85] Luria S. E., and Delbrück M. (1943) ‘Mutations of Bacteria from Virus Sensitivity to Virus Resistance’, *Genetics*, 28: 491–511. 17247100 10.1093/genetics/28.6.491 PMC1209226

[R86] Maffei E. et al. (2022) ‘Phage Paride Hijacks Bacterial Stress Responses to Kill Dormant, Antibiotic-Tolerant Cells’, *BioRxiv*.

[R87] Maimaiti Z. et al. (2023) ‘Global Trends and Hotspots of Phage Therapy for Bacterial Infection: A Bibliometric Visualized Analysis from 2001 to 2021’, *Frontiers in Microbiology*, 13: 1067803. 10.3389/fmicb.2022.1067803 PMC9868171 36699585

[R88] Mangalea M. R., Duerkop B. A., and Ottemann K. M. (2020) ‘Fitness Trade-Offs Resulting from Bacteriophage Resistance Potentiate Synergistic Antibacterial Strategies’, *Infection and Immunity*, 88: e00926–19. 32094257 10.1128/IAI.00926-19 PMC7309606

[R89] Manrique P. et al. (2016) ‘Healthy Human Gut Phageome’, *Proceedings of the National Academy of Sciences*, 113: 10400–5. 10.1073/pnas.1601060113 PMC5027468 27573828

[R90] Matsuura M. (2013) ‘Structural Modifications of Bacterial Lipopolysaccharide That Facilitate Gram-Negative Bacteria Evasion of Host Innate Immunity’, *Frontiers in Immunology*, 4: 109. 10.3389/fimmu.2013.00109 PMC3662973 23745121

[R91] Mattila S., Ruotsalainen P., and Jalasvuori M. (2015) ‘On-Demand Isolation of Bacteriophages against Drug-Resistant Bacteria for Personalized Phage Therapy’, *Frontiers in Microbiology*, 6: 1271. 10.3389/fmicb.2015.01271 PMC4643220 26617601

[R92] Mayr U. B. et al. (2005) ‘Bacterial Ghosts as Antigen Delivery Vehicles’, *Advanced Drug Delivery Reviews*, 57: 1381–91. 15878634 10.1016/j.addr.2005.01.027

[R93] McCallin S. et al. (2018) ‘Metagenome Analysis of Russian and Georgian Pyophage Cocktails and a Placebo-Controlled Safety Trial of Single Phage versus Phage Cocktail in Healthy *Staphylococcus aureus* Carriers’, *Environmental Microbiology*, 20: 3278–93. 30051571 10.1111/1462-2920.14310

[R94] McCallin S. et al. (2019) ‘Current State of Compassionate Phage Therapy’, *Viruses*, 11: 343. 10.3390/v11040343 PMC6521059 31013833

[R95] Meyer J. R. et al. (2012) ‘Repeatability and Contingency in the Evolution of a Key Innovation in Phage Lambda’, *Science*, 335: 428–32. 22282803 10.1126/science.1214449 PMC3306806

[R96] Michel A. et al. (2010) ‘Bacteriophage PhiX174’s Ecological Niche and the Flexibility of Its *Escherichia coli* Lipopolysaccharide Receptor’, *Applied and Environmental Microbiology*, 76: 7310–3. 20833781 10.1128/AEM.02721-09 PMC2976268

[R97] Międzybrodzki R. et al. (2018) ‘Current Updates from the Long-Standing Phage Research Centers in Georgia, Poland, and Russia’, in Harper, D. R. et al. (eds) *Bacteriophages: Biology, Technology, Therapy*, pp. 1–31. Springer International Publishing: Cham.

[R98] Mu A. et al. (2021) ‘Assessment of the Microbiome during Bacteriophage Therapy in Combination with Systemic Antibiotics to Treat a Case of Staphylococcal Device Infection’, *Microbiome*, 9: 1–8. 33853672 10.1186/s40168-021-01026-9 PMC8048313

[R99] Mukherjee S. et al. (2015) ‘Large-Scale Contamination of Microbial Isolate Genomes by Illumina PhiX Control’, *Standards in Genomic Sciences*, 10: 1–4. 26203331 10.1186/1944-3277-10-18 PMC4511556

[R100] Mulani M. S. et al. (2019) ‘Emerging Strategies to Combat ESKAPE Pathogens in the Era of Antimicrobial Resistance: A Review’, *Frontiers in Microbiology*, 10: 539. 10.3389/fmicb.2019.00539 PMC6452778 30988669

[R101] Murray C. J. et al. (2022) ‘Global Burden of Bacterial Antimicrobial Resistance in 2019: A Systematic Analysis’, *The Lancet*, 399: 629–55. 10.1016/S0140-6736(21)02724-0 PMC8841637 35065702

[R102] Mutalik V. K. et al. (2020) ‘High-Throughput Mapping of the Phage Resistance Landscape in *E. coli*’, *PLoS Biology*, 18: e3000877. 10.1371/journal.pbio.3000877 PMC7553319 33048924

[R103] Mutti M., and Corsini L. (2019) ‘Robust Approaches for the Production of Active Ingredient and Drug Product for Human Phage Therapy’, *Frontiers in Microbiology*, 10: 2289. 10.3389/fmicb.2019.02289 PMC6791927 31649636

[R104] Nale J. Y. et al. (2021) ‘An Optimized Bacteriophage Cocktail Can Effectively Control *Salmonella in Vitro* and in *Galleria mellonella*’, *Frontiers in Microbiology*, 11: 609955. 10.3389/fmicb.2020.609955 PMC7858669 33552020

[R105] Niang M. et al. (2021) ‘Why Is Repositioning Public Health Innovation towards a Social Paradigm Necessary? A Reflection on the Field of Public Health through the Examples of Ebola and Covid-19’, *Globalization & Health*, 17: 1–11. 33853631 10.1186/s12992-021-00695-3 PMC8045578

[R106] Ochs H. D., Davis S. D., and Wedgwood R. J. (1971) ‘Immunologic Responses to Bacteriophage ΦX174 in Immunodeficiency Diseases’, *The Journal of Clinical Investigation*, 50: 2559–68. 5129308 10.1172/JCI106756 PMC292205

[R107] Orta A. K. et al. (2023) ‘The Mechanism of the Phage-Encoded Protein Antibiotic from ΦX174’, *Science (New York, N.Y.)*, 381: eadg9091. 10.1126/science.adg9091 PMC12747129 37440661

[R108] Pagnout C. et al. (2019) ‘Pleiotropic Effects of *Rfa*-Gene Mutations on *Escherichia coli* Envelope Properties’, *Scientific Reports*, 9: 9696. 10.1038/s41598-019-46100-3 PMC6609704 31273247

[R109] Parab L. et al. (2023) ‘Chloramphenicol Reduces Phage Resistance Evolution by Suppressing Bacterial Cell Surface Mutants’, *BioRxiv*.

[R110] Pirnay J.-P. et al. (2015) ‘Quality and Safety Requirements for Sustainable Phage Therapy Products’, *Pharmaceutical Research*, 32: 2173–9. 25585954 10.1007/s11095-014-1617-7 PMC4452253

[R111] Pirnay J.-P. et al. (2018) ‘The Magistral Phage’, *Viruses*, 10: 64. 10.3390/v10020064 PMC5850371 29415431

[R112] Ramirez J. et al. (2020) ‘Antibiotics as Major Disruptors of Gut Microbiota’, *Frontiers in Cellular & Infection Microbiology*, 10: 572912. 10.3389/fcimb.2020.572912 PMC7732679 33330122

[R113] Rao V. B., and Zhu J. (2022) ‘Bacteriophage T4 as a Nanovehicle for Delivery of Genes and Therapeutics into Human Cells’, *Current Opinion in Virology*, 55: 101255. 10.1016/j.coviro.2022.101255 PMC11736861 35952598

[R114] Rios A. C. et al. (2016) ‘Alternatives to Overcoming Bacterial Resistances: State-of-the-Art’, *Microbiological Research*, 191: 51–80. 27524653 10.1016/j.micres.2016.04.008

[R115] Roach D. R. et al. (2017) ‘Synergy between the Host Immune System and Bacteriophage Is Essential for Successful Phage Therapy against an Acute Respiratory Pathogen’, *Cell Host & Microbe*, 22: 38–47.e4. 28704651 10.1016/j.chom.2017.06.018

[R116] Rohde C. et al. (2018) ‘Expert Opinion on Three Phage Therapy Related Topics: Bacterial Phage Resistance, Phage Training and Prophages in Bacterial Production Strains’, *Viruses*, 10: 178. 10.3390/v10040178 PMC5923472 29621199

[R117] Romeyer Dherbey J. (2023), Evolutionary Exploration of a Bacterial LPS Genotype to Phenotype Map with Phages <https://macau.uni-kiel.de/receive/macau_mods_00003546> accessed 24 Jan 2024.

[R118] Romeyer Dherbey J. et al. (2023) ‘Stepwise Evolution of *E. coli* C and ΦX174 Reveals Unexpected Lipopolysaccharide (LPS) Diversity’, *Molecular Biology and Evolution*, 40: msad154. 10.1093/molbev/msad154 PMC10368449 37399035

[R119] Roznowski A. P. et al. (2019) ‘Recessive Host Range Mutants and Unsusceptible Cells That Inactivate Virions without Genome Penetration: Ecological and Technical Implications’, *Journal of Virology*, 93: 10–128. 10.1128/JVI.01767-18 PMC6340031 30429341

[R120] Rubinstein A. et al. (2000) ‘Progressive Specific Immune Attrition after Primary, Secondary and Tertiary Immunizations with Bacteriophage ΦX174 in Asymptomatic HIV-1 Infected Patients’, *AIDS (London, England)*, 14: F55–62. 10770533 10.1097/00002030-200003100-00004

[R121] Russell P. W., and Müller U. R. (1984) ‘Construction of Bacteriophage Luminal Diameter ΦX174 Mutants with Maximum Genome Sizes’, *Journal of Virology*, 52: 822–7. 6092714 10.1128/jvi.52.3.822-827.1984 PMC254601

[R122] Sanger F. et al. (1978) ‘The Nucleotide Sequence of Bacteriophage ΦX174’, *Journal of Molecular Biology*, 125: 225–46. 731693 10.1016/0022-2836(78)90346-7

[R123] Sausset R. et al. (2020) ‘New Insights into Intestinal Phages’, *Mucosal Immunology*, 13: 205–15. 31907364 10.1038/s41385-019-0250-5 PMC7039812

[R124] Schooley R. T. et al. (2017) ‘Development and Use of Personalized Bacteriophage-Based Therapeutic Cocktails to Treat a Patient with a Disseminated Resistant *Acinetobacter baumannii* Infection’, *Antimicrobial Agents and Chemotherapy*, 61: e00954–17. 28807909 10.1128/AAC.00954-17 PMC5610518

[R125] Science, Innovation, and Technology Committee . (2023), The Antimicrobial Potential of Bacteriophages <https://committees.parliament.uk/event/17021/formal-meeting-oral-evidence-session/> accessed 24 Jan 2024.

[R126] Sertic V., and Bulgakov N. (1935) ‘Sertic & Boulgakov 1935 Classification Et Identification Des Typhi-Phages’, *Comptes Rendus de Societe Biologique* (*Paris*), 119: 1270–2.

[R127] Shkoporov A. N. et al. (2019) ‘The Human Gut Virome Is Highly Diverse, Stable, and Individual Specific’, *Cell Host & Microbe*, 26: 527–41.e5. 31600503 10.1016/j.chom.2019.09.009

[R128] Simpson B. W., and Trent M. S. (2019) ‘Pushing the Envelope: LPS Modifications and Their Consequences’, *Nature Reviews, Microbiology*, 17: 403–16. 31142822 10.1038/s41579-019-0201-x PMC6913091

[R129] Sinsheimer R. L. (1959) ‘Purification and Properties of Bacteriophage ΦX174’, *Journal of Molecular Biology*, 1: 37–42. 10.1016/s0022-2836(63)80017-0 13978804

[R130] Skaradzińska A. et al. (2020) ‘Bacteriophage Amplification—A Comparison of Selected Methods’, *Journal of Virological Methods*, 282: 113856. 10.1016/j.jviromet.2020.113856 32198027

[R131] Smith H. O. et al. (2003) ‘Generating a Synthetic Genome by Whole Genome Assembly: ΦX174 Bacteriophage from Synthetic Oligonucleotides’, *Proceedings of the National Academy of Sciences of the United States of America*., 100: 15440–5. 14657399 10.1073/pnas.2237126100 PMC307586

[R132] Srikant S., Guegler C. K., and Laub M. T. (2022) ‘The Evolution of a Counter-Defense Mechanism in a Virus Constrains Its Host Range’, *eLife*, 11: e79549. 10.7554/eLife.79549 PMC9391042 35924892

[R133] Staub N. L. et al. (2016) ‘Scaling Up: Adapting a Phage-Hunting Course to Increase Participation of First-Year Students in Research’, *CBE Life Sciences Education*, 15: ar13. 10.1187/cbe.15-10-0211 PMC4909335 27146160

[R134] Streicher L. M. (2021) ‘Exploring the Future of Infectious Disease Treatment in a Post-Antibiotic Era: A Comparative Review of Alternative Therapeutics’, *Journal of Global Antimicrobial Resistance*, 24: 285–95. 33484895 10.1016/j.jgar.2020.12.025

[R135] Summers W. C. (2012) ‘The Strange History of Phage Therapy’, *Bacteriophage*, 2: 130–3. 23050223 10.4161/bact.20757 PMC3442826

[R136] Tabib-Salazar A. et al. (2018) ‘T7 Phage Factor Required for Managing RpoS in *Escherichia coli*’, *Proceedings of the National Academy of Sciences*, 115: E5353–62. 10.1073/pnas.1800429115 PMC6003314 29789383

[R137] Tisza M. J. et al. (2020) ‘Discovery of Several Thousand Highly Diverse Circular DNA Viruses’, *eLife*, 9: e51971. 10.7554/eLife.51971 PMC7000223 32014111

[R138] Villarroel J. et al. (2017) ‘Metagenomic Analysis of Therapeutic PYO Phage Cocktails from 1997 to 2014’, *Viruses*, 9: 328. 10.3390/v9110328 PMC5707535 29099783

[R139] Weber-Dąbrowska B. et al. (2016) ‘Bacteriophage Procurement for Therapeutic Purposes’, *Frontiers in Microbiology*, 7: 1177. 10.3389/fmicb.2016.01177 PMC4981656 27570518

[R140] WHO . (2021), Antimicrobial Resistance <https://www.who.int/news-room/fact-sheets/detail/antimicrobial-resistance> accessed 24 Jan 2024.

[R141] Wichman H. A., and Brown C. J. (2010) ‘Experimental Evolution of Viruses: *Microviridae* as a Model System’, *Philosophical Transactions of the Royal Society B: Biological Sciences*, 365: 2495–501. 10.1098/rstb.2010.0053 PMC2935103 20643739

[R142] Wichman H. A., Millstein J., and Bull J. J. (2005) ‘Adaptive Molecular Evolution for 13,000 Phage Generations: A Possible Arms Race’, *Genetics*, 170: 19–31. 15687276 10.1534/genetics.104.034488 PMC1449705

[R143] Yang Y. et al. (2020) ‘Development of a Bacteriophage Cocktail to Constrain the Emergence of Phage-Resistant *Pseudomonas aeruginosa*’, *Frontiers in Microbiology*, 11: 327. 10.3389/fmicb.2020.00327 PMC7065532 32194532

[R144] Yehl K. et al. (2019) ‘Engineering Phage Host-Range and Suppressing Bacterial Resistance through Phage Tail Fiber Mutagenesis’, *Cell*, 179: 459–69.e9. 31585083 10.1016/j.cell.2019.09.015 PMC6924272

[R145] Yethon J. A. et al. (2000) ‘Mutation of the Lipopolysaccharide Core Glycosyltransferase Encoded by waaG Destabilizes the Outer Membrane of Escherichia coli by Interfering with Core Phosphorylation’, *Journal of Bacteriology*, 182: 5620–3. 10986272 10.1128/jb.182.19.5620-5623.2000 PMC111012

[R146] Yoichi M. et al. (2005) ‘Alteration of Tail Fiber Protein Gp38 Enables T2 Phage to Infect Escherichia coli O157:H7’, *Journal of Biotechnology*, 115: 101–7. 15607229 10.1016/j.jbiotec.2004.08.003

[R147] Yu L. X. et al. (2014) ‘Understanding Pharmaceutical Quality by Design’, *The AAPS Journal*, 16: 771–83. 24854893 10.1208/s12248-014-9598-3 PMC4070262

[R148] Zhu J. et al. (2023) ‘Design of Bacteriophage T4-Based Artificial Viral Vectors for Human Genome Remodeling’, *Nature Communications*, 14: 2928. 10.1038/s41467-023-38364-1 PMC10229621 37253769

[R149] Zhvania P. et al. (2017) ‘Phage Therapy in a 16-Year-Old Boy with Netherton Syndrome’, *Frontiers in Medicine*, 4: 94. 10.3389/fmed.2017.00094 PMC5494523 28717637

[R150] Zuo T. et al. (2020) ‘Human-Gut-DNA Virome Variations across Geography, Ethnicity, and Urbanization’, *Cell Host & Microbe*, 28: 741–51.e4. 32910902 10.1016/j.chom.2020.08.005

